# Gross-Motor Coordination and Executive Functions Development in Soccer and Artistic Gymnastics Preadolescent Female Athletes

**DOI:** 10.3390/jfmk10010085

**Published:** 2025-03-01

**Authors:** Fioretta Silvestri, Matteo Campanella, Lorenzo Marcelli, Dafne Ferrari, Maria Chiara Gallotta, Faten Hamdi, Maicon Rodrigues Albuquerque, Maurizio Bertollo, Davide Curzi

**Affiliations:** 1 Department of Humanities, Movement and Education Sciences, University “Niccolò Cusano”, 00166 Rome, Italy; fioretta.silvestri@unicusano.it; 2 Department of Theoretical and Applied Sciences, eCampus University, 22060 Novedrate, Italy; matteo.campanella93@gmail.com; 3 Department of Life Sciences, Health and Health Professions, Link Campus University, 00165 Rome, Italy; d.ferrari@unilink.it; 4 Department of Physiology and Pharmacology “Vittorio Erspamer”, Sapienza University of Rome, 00185 Rome, Italy; mariachiara.gallotta@uniroma1.it; 5 High Institute of Sport and Physical Education of Kef, University of Jendouba, El Kef 7100, Tunisia; faten.hamdi@issepkef.u-jendouba.tn; 6 Neurosciences of Physical Activity and Sports Research Group, Department of Sports, Universidade Federal de Minas Gerais, Belo Horizonte 31120-901, Brazil; lin.maicon@gmail.com; 7 BIND-Behavioral Imaging and Neural Dynamics Center, Department of Medicine and Aging Sciences, University G. d’Annunzio of Chieti-Pescara, 66100 Chieti, Italy; m.bertollo@unich.it

**Keywords:** cognitive functions, KTK, inhibitory control, working memory, open-skill sport, closed-skill sport, expertise level

## Abstract

**Background/Objectives**: The characteristics of open- and closed-skill sports can have a significant impact on the development of both motor and cognitive functions during late childhood and early adolescence. This study examined the impact of artistic gymnastics and soccer practice on physical/coordinative skills and executive functions in preadolescent female athletes. **Methods**: Gross-motor coordination (Körperkoordinations Test für Kinder battery), explosive lower limb power (countermovement jump) and executive functions (Flanker/Reverse Flanker; Digit Span) of ninety-eight soccer and gymnastics athletes (10.9 ± 0.6 years of age) were tested at the beginning and the end of a sports season. A *t*-test and a mixed linear method were used to detect differences between sports and expertise levels (amateur vs. elite) at baseline and over time, respectively. **Results**: Gymnasts scored higher in physical tests compared to soccer athletes, who obtained better scores in the Digit Span test. Elite gymnasts showed higher physical skills compared to both amateur gymnasts and elite soccer players, with executive function levels comparable to those of amateur gymnasts. Similarly, elite soccer players showed higher reaction time in executive functions tests compared to both elite gymnasts and amateur soccer athletes, with similar physical skills as amateur soccer players. Amateur gymnastics and soccer athletes showed similar levels of physical and cognitive skills. All groups improved their physical and executive function skills over time, with relevant effects of expertise level on physical tasks. **Conclusions**: Results revealed the impact of sport type and athletes’ level of expertise in influencing both their motor and cognitive development.

## 1. Introduction

The development of both motor and cognitive functions during growth has been widely associated with physical activity (PA) practice [1,2]. Gross-motor coordination (GMC) is the ability to perform a range of fundamental motor skills, including activities such as running, jumping, and hopping [3]. It reaches its maximal improvement rate during late childhood (8–10 years of age) and stabilizes during preadolescence (10 to 13 years of age) even if not linearly [2]. Although the positive association between GMC and PA practice is quite apparent, recent studies have also focused on its connection with the development of executive functions (EFs) [4,5]. Inhibitory control (IC), working memory (WM) and cognitive flexibility, also defined as the three core EFs [6], are foundational to cognitive processes such as intentional control and decision-making, which are essential components of sports practice. The association between motor development and EFs may be particularly strong in younger children [7]. This suggests that early motor development may serve as a catalyst for cognitive enhancement, reinforcing the importance of structured interventions during early childhood. Furthermore, the involvement of neural mechanisms shared by motor and cognitive processes emphasizes the potential benefits of specific sports practices, particularly those requiring complex, adaptive movements [8]. Additionally, recent studies have positively correlated children’s sport participation with a higher level of EFs [9], showing that both type and frequency of PA are predictors of children’s EFs development [4]. In this scenario, researchers are now studying the effects of different types of sport interventions on both motor coordination development [10] and cognitive performance [11,12]. Recent reviews revealed that organized sport is linked to EFs development [9] and that open-skill sports (OSS) practice is superior in improving some aspects of cognitive functions compared with closed-skill sports (CSS) [13,14]. OSS involve dynamic environments where participants must adjust to unpredictable external stimuli (e.g., soccer, tennis, basketball), while CSS are carried out in stable, predictable settings and athletes are required to follow predefined movement patterns (e.g., swimming, track and field) [13]. However, it has been suggested that the heightened engagement of executive functions is more strongly associated with a sport’s cognitive demands than with the simple distinction between open- and closed-skill activities [14]. In fact, factors such as object-control skills [15], whether the sport is individual or team-based [16], the type of movement (cyclic or variable) [17], and the presence of an indoor or outdoor environment [18] could all influence both motor and cognitive domains to varying extents. For these reasons, there is still scarce evidence to suggest the preference of one sport modality over others [9].

Formenti et al. [19] found that individuals practicing OSS demonstrated higher levels of both EFs (IC) and motor capabilities (speed, agility and power) compared to those who have been practicing CSS. The authors suggest that the complexity and variability of the environment, which are characteristics of OSS, play a key role in the development of both motor and cognitive components in children. However, other authors have highlighted that the distinction between these categories of sports is not so clear-cut. For instance, in their 12 weeks of interventional study, Contreras-Osorio et al. [12], found that both groups of children practicing open-skill (handball) and closed-skill (athletics) sports improved their motor skills and EFs over time, compared to a control group who did not engage in any PA program. Additionally, Spanou et al. [16] analysed 115 children (8–12 years of age) practicing OSS and CSS, finding that the involvement in different types of sports differentiates their motor competence (MC) but not their EFs. Specifically, children who practiced gymnastics and track and field, showed higher levels of MC compared to participants practicing open-skill team (volleyball, football) or individual sports (tennis), with similar levels of IC and cognitive flexibility [16].

Within the CSS, technical-combinatory sports, like gymnastics, require high body control. In the past decades, it has been observed that motor control and EFs share common regions of brain activity, such as the prefrontal cortex and the cerebellum [8]. This interrelation could positively affect both GMC and EFs development in children practicing technical-combinatory sports. Research has shown that 8–10-year-old gymnasts have a higher level of GMC compared to other CSS, like swimming or cycling, when there is a cyclical repetition of one sport-specific movement [10]. In addition, participation in balance sports such as artistic gymnastics requires complex bodily movement adjustments in space and an effective integration of proprioceptive, visual and vestibular information, that could reflect in higher spatial cognitive functions in these athletes, likely based on common neural circuits [20]. Moreover, young and preadolescent gymnasts overperformed both soccer athletes and a control group in a verbal [1] and spatial WM task [21], respectively.

However, these positive effects found in gymnastics athletes could also depend on the time spent doing physical exercise and on the individual’s expertise. The early specialization leads young gymnasts to cope with a high amount of training hours compared to peers practicing other closed- and OSS with later specialization [22]. Thus, in the analysis of the sport interventions that could benefit both coordinative and cognitive areas, it is also important to consider the level of expertise. Elite or amateur athletes, despite the sport type, are involved in a considerably different weekly training volume and intensity of training, which could be a crucial factor for both cognitive and motor growth. Verburgh et al. [23] analysed 168 preadolescents involved in amateur or professional soccer activity and found that the time spent playing was significantly associated with a better performance in IC, WM and attention. However, other authors found no significant effects of expertise level in adolescent athletes playing both open- and CSS on decision-making, WM and IC [24]. Thus, although there is general agreement that the athlete’s level is highly correlated with improvements in GMC [10,11,12,13,14,15,16,17,18,19,20,21,22,23,24,25], fewer conclusive results exist regarding EFs enhancement. Therefore, it is essential to also understand how the athletes’ expertise level might affect these outcomes and to what extent it could influence motor and cognitive development in late childhood.

In summary, it seems necessary to provide additional evidence on the physical and cognitive functions of preadolescent athletes practicing sports with dissimilar motor and cognitive demands and at different levels of expertise. Moreover, there is a lack of longitudinal studies in this field, as the cited studies are mainly cross-sectional, observational or retrospective. Thus, the present study aims to explore the impact of practicing open- or CSS on motor skills and EFs development in preadolescent female athletes. Specifically, it seeks to examine these two components in athletes who play soccer—an OSS with high variability of stimuli and opponents’ presence—and athletes who practice artistic gymnastics—a technical-combinatory CSS—at different levels of expertise. Additionally, the study aims to investigate the correlation between physical skills and EFs in this population. This study hypothesizes that athletes practicing OSS would exhibit higher levels of cognitive skills, whereas those practicing CSS would perform better in physical tests. Additionally, we hypothesize that the athletes’ level of expertise would positively impact both motor and cognitive skills.

## 2. Materials and Methods

### 2.1. Participants

Ninety-eight preadolescent female athletes were included in the study. Forty-four subjects practicing artistic gymnastics (mean age 10.6 ± 0.6 years, height 144.7 ± 7.5 cm, weight 37.9 ± 7.9 kg, BMI 19.9 ± 2.2 kg/m^2^) were recruited from amateur (n = 18) and elite (n = 26) groups of an Italian gymnastics club (A.S.D. Ginnastica Roma Aurelio), while fifty-four soccer athletes (mean age 11.1 ± 0.5 years, height 148.5 ± 7.5 cm, weight 44.3 ± 11.2 kg, BMI 19.9 ± 4.0 kg/m^2^) were recruited from an elite (n = 21—SS Lazio Women) and an amateur (n = 33—Roma Calcio Femminile) Italian soccer club. All athletes voluntarily participated in the research. To be eligible, participants have to have accomplished the following requirements:

- To be healthy and in the absence of any physical or psychological issues that might affect the study.
- To have at least two years of gymnastics/soccer training experience.
- To have been regularly attending the seasonal trainings and competitions.

This research was evaluated and authorized by the Institutional Review Board of the University Niccolò Cusano, study protocol number MO 8/22, in accordance with the 1964 Helsinki Declaration and its subsequent revisions or equivalent ethical standards. Participants and their parents received detailed information about the study, including a written document, and provided their informed consent in writing prior to their involvement in the research.

### 2.2. Study Design

**Figure 1:**
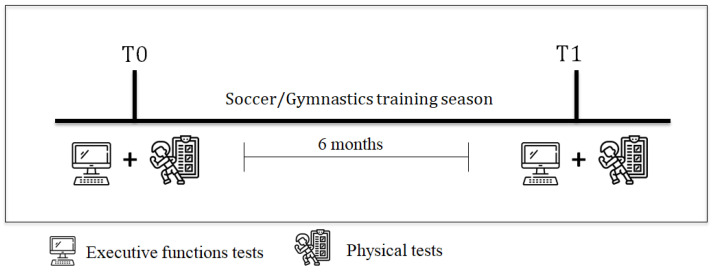
Study design timeline.

All participants were evaluated at the beginning (T0—after the preparation phase) and at the end (T1—6 months after) of a training/competitive youth gymnastics or soccer season (Figure 1). The testing protocol started at 5:30 p.m. with the cognitive assessment (approximately 10 min EFs computer-based tests), followed by the physical assessment (motor tests) to avoid the effects of physical fatigue on cognitive capabilities. During the training season, gymnastics athletes practiced indoor training, composed of a warm up–conditioning phase, followed by a phase of technical training aimed at competition preparation. Soccer athletes played outdoor training, composed of a general warm up and conditioning phase, technical-tactical training and soccer matches. Elite athletes distinguished themselves from amateur athletes by being selected by their respective clubs for the elite team and by engaging in a higher weekly training volume [26] compared to their amateur counterparts (Table 1).

### 2.3. Executive Functions Assessment

The computer version of the Flanker/Reverse Flanker Task was used to evaluate inhibition and cognitive flexibility. This test was chosen because it is considered a reliable test for EFs assessment and should be administered without a preliminary training session [27]. Moreover, it has already been utilized in studies evaluating EFs skills involving young athletes across various sports disciplines [28,29].

It consisted of three blocks, each displaying a sequence of five fish, either blue or pink. The first block followed the traditional Flanker paradigm [30], with all five fish blue. Participants focused on the central fish and indicated its direction by pressing the corresponding key (left or right) while ignoring the flanking fish. In the second block, a Reverse Flanker condition was introduced, where all five fish were pink. Here, participants ignored the central fish and responded based on the direction of the fish at the two ends. The third block was a mixed condition, with fish colours alternating randomly between blue and pink. Participants applied the same rules from the previous blocks, depending on the fish colour, testing their ability to switch between rules and engage cognitive control processes like attention, inhibition, reorientation, and rule retention. Each of the first two blocks included 22 trials (16 congruent and 6 incongruent), and the third block had 44 trials (32 congruent and 12 incongruent), totalling 88 trials. Familiarization trials were conducted before each block and were excluded from analysis, which included only data from the third block. Accuracy (Flank_Acc: % of correct responses) and average response time (Flank_RT) were used for data analysis.

The Forward- and Backward-Digit Span tests were used to assess WM [6,7,8,9,10,11,12,13,14,15,16,17,18,19,20,21,22,23,24,25,26,27,28,29,30,31]. This test is one of the most widely used assessments of WM in research [32] and has been used in studies on exercise and cognition in children [33] and in adolescent athletes [31].

Participants viewed a sequence of digits on a computer screen, presented at a rate of one digit per second. They were asked to input the digits in the same order for the Forward-Digit Span or in reverse order for the Backward-Digit Span. If they correctly recalled the sequence, its length was increased by one digit, continuing until they failed to recall two consecutive sequences of the same length. The participant’s digit span was the longest sequence they could accurately recall, which provides the direct (Span_for) or the reverse (Span_back) Span score (first outcome). In addition, the Reaction Time (Span_for_RT and Span_back_RT) and the Rate of Correct Score (Span_RCS: Span Score/Span_RT), were calculated for both forward (Span_for_RCS) and backward tests (Span_back_RCS) and used for further analyses.

### 2.4. Motor Performance Assessment

In order to assess the physical skills of gymnasts and soccer players both GMC and explosive lower limb power were tested, with tasks involving balance, speed, agility and explosive power, according to the previous literature [19].

GMC was assessed using the Körperkoordinations Test für Kinder (KTK) battery [34], which has a test–retest reliability coefficient of 0.97 [35]. The KTK is widely used as a measure of motor competence for various research purposes involving children in diverse contexts. It includes non-sport/skill-specific tasks and enables cross-study comparisons [36]. Moreover, it has been used in studies involving sports populations aged 8–11 years [2,3,4,5,6,7,8,9,10,11,12,13,14,15,16,17,18,19,20,21,22,23,24,25,26,27,28,29,30,31,32,33,34,35,36,37].

All participants followed some familiarization trials before starting each test, after an oral explanation and practical demonstration provided by the scientific staff. The KTK battery was composed of the following four tests:

The Balance Beam (BB) test evaluates balance control and coordination by progressively narrowing the walking surface. Participants are required to step backward three times across three balance beams, each measuring 3 m in length and 8 cm in height. The width of the beams reduces as the test progresses, with widths set at 6.0 cm, 4.5 cm, and 3.0 cm for the three stages. A maximum of eight steps per trial is permitted on each beam, leading to a cumulative total of 72 steps (8 steps × 3 trials × 3 beams). The participant’s score reflects the total number of steps completed across all trials.

The Monopodalic Jump (MJ) test assesses lower limb power, coordination, and dynamic stability. Participants were required to run up (about 1.5 m) and jump on one leg over a stack of foam mats (5 cm high, 60 cm wide, and 20 cm deep), with the other leg remaining off the ground. The initial mat height was set at 25 cm. Points are awarded for successful jumps on the first (3 points), second (2 points), or third (1 point) attempt. For each leg, each successful jump adds a foam mat, and the test ends after three failures with the same leg. The final score was calculated by the total number of foam mats successfully cleared. The maximum possible score for each leg was 39 points with a maximum total score of 78 points.

The Lateral Jump (LJ) test measures speed, dynamic balance and bilateral coordination. Participants were required to jump horizontally using both feet over a rectangular mat with a wooden slat (60 × 4 × 2 cm) dividing it into two identical squares. Two 15-s trials were performed, within which the participants had to execute as many jumps as possible. The final score was determined by the total number of jumps completed across both trials.

The Plate Shift (PS) test, evaluates the agility of lateral movements, integrating upper and lower limb coordination. Participants stooped on one platform (25 × 25 × 5.7 cm) and were required to move the adjacent platform laterally, using both hands, and shift onto it as rapidly as possible. Each participant performed two 20-s trials following the preferred direction. The final score was the sum of the steps in the two trials.

The total KTK test score (sum of the four tests) was converted into a Motor Quotient value (MQ), which is age- and gender-specific (based on normalisation tables) and reflects the level of GMC, with a mean of 100 and a normal range between 85 and 115 points [35,36].

In addition to the KTK battery, which is limited to balance and locomotor skills [36], the countermovement jump (CMJ) was used to assess explosive lower limb power, which may also be related to cognitive performance in children [38] and has been used in previous studies assessing the interaction between EFs and MC in this population [19].

From a vertical standing position, participants were asked to perform a countermovement (reaching approximately 90° of knee flexion) and, as quickly as possible, to execute a maximal vertical jump with arms blocked at the hips. A total of three correct jumps was performed using an optical measurement system (Optojump, Microgate, Udine, Italy—1.0416 cm resolution) which has been demonstrated to be a valid tool for CMJ-derived parameters assessment. The maximal jump height over the three attempts was calculated and used as the outcome, as it is the main indicator of a vertical jump performance [39].

### 2.5. Statistical Analysis

Statistical analysis was performed using IBM SPSS statistics 23.0. After checking for normal distribution, the student *t*-test and the Mann–Whitney test (for variables which did not follow a normal distribution: Flank_acc, Span score, Span RT), were used to detect baseline differences between groups practicing different sports (Gymnastics vs. Soccer) and between athletes of different levels (elite vs. amateur) practicing the same sport at T0. To interpret the data, effect sizes were calculated using Cohen’s d for the aforementioned tests, defined as small (d = 0.2), medium (d = 0.5), and large (d = 0.8) effect sizes [40]. A mixed linear method with time (T0 vs. T1), sport (Gymnastics vs. Soccer) and level (elite vs amateur) as fixed factors, and subjects as random factors, was used to detect the changes in the assessed parameter over time in the four groups. In line with other studies [27], partial eta-squared (ηp^2^) was employed as a measure of effect size and categorized according to the following scale: small (<0.09); medium (0.09 to <0.25); large (≥0.25). Finally, the Spearman correlation coefficient (ρ) was used to describe the relationship between motor and cognitive data of the whole sample. G*power analysis revealed that a total sample of ninety-eight subjects would be sufficient to estimate medium to large effect size (baseline differences) and medium effect size (mixed linear method) with a statistical power of 0.8. The significance was set at 0.05. Data were reported as mean and standard deviation.

## 3. Results

In Table 2, anthropometric data (weight, height and BMI) of the four groups are reported. Elite soccer athletes showed significantly higher weight and BMI compared to elite gymnasts. No differences were observed between the other groups.

### 3.1. Baseline Differences by Sports

At baseline, the groups showed significant differences in several tests of the KTK battery. Gymnastics athletes showed better scores than soccer athletes in three out of four GMC tests. In particular, they showed higher BB (54.4 ± 11.3 score vs. 43.6 ± 11.3 score; *t* = 4.72, *p* < 0.001, d = 0.96), LJ (66.0 ± 8.4 score vs. 60.9 ± 11.9 score; *t* = 2.35, *p* = 0.021, d = 0.50), MJ (54.6 ± 11.8 score vs. 47.7 ± 11.4 score; *t* = 2.90, *p* = 0.005, d = 0.59) and total MQ score (93.8 ± 9.8 score vs. 82.8 ± 11.4 score; *t* = 4.96, *p* < 0.001, d = 1.03) compared to the soccer group. No differences in the PS test were observed between the two sports. In the height of the CMJ, the gymnastics group showed significantly better scores than the soccer group (22.4 ± 4.4 cm vs. 19.8 ± 4.0 cm; *t* = 3.03, *p* = 0.003, d = 0.62). Comparing EFs parameters, no significant differences were observed in baseline Flanker and Digit span test scores between groups practicing different sports, except for the Span_for_RT, where soccer athletes showed significantly better scores compared to gymnastics athletes (2700.1 ± 539.6 ms vs. 3209.1 ± 1403.8 ms; *p* = 0.05, d = 0.48).

### 3.2. Baseline Differences by Level of Expertise

Comparing gymnastics athletes of different levels, the results showed significantly better scores of elite gymnastics athletes in BB (*t* = 3.03, *p* = 0.004, d = 0.95), MJ (*t* = 2.97, *p* = 0.005, d = 0.92) and a tendency to show a significantly better total MQ score (*t* = 1.98, *p* = 0.054, d = 0.61) compared to the amateur gymnastics group. However, comparing the Flanker and Span Digit tests, no differences were detected in any of the measured EFs parameters between the gymnastic groups. Analysing soccer athletes of different levels, the results showed similar values of motor coordination (MQ and CMJ) except for MJ, where amateur soccer athletes showed better scores than elite athletes (*t* = 2.95, *p* = 0.005, d = 0.86). However, elite soccer athletes showed significantly better results in the RT of both Flanker (*t* = 2.24, *p* = 0.03, d = 0.60) and Backward-Span Digit (*p* = 0.014, d = 0.41) tests compared to the amateur soccer group. No differences were detected between soccer groups in the other EFs parameters.

In addition, when comparing only elite groups, gymnastics athletes showed better BB (*t* = 4.89, *p* < 0.001, d = 1.42), MJ (t = 5.85, *p* < 0.001, d = 1.75), total MQ score (t = 5.68, *p* < 0.001, d = 1.67) and CMJ (t = 2.61, *p* = 0.012, d = 0.77) compared to elite soccer athletes, while elite soccer athletes showed better RT in both Flanker (t = 2.05, *p* = 0.047, d = 0.60) and Backward-Digit Span test (*p* = 0.038, d = 0.22). No differences in cognitive or motor performance were observed when comparing amateur athletes of different sports (except for LJ which was greater in the gymnastics group: *t* = 2.10, *p* = 0.041, d = 0.65).

The mean and standard deviations baseline values of both motor and cognitive tests of the four groups are shown in Table 3.

### 3.3. Motor Performance Differences over Time

**Figure 2:**
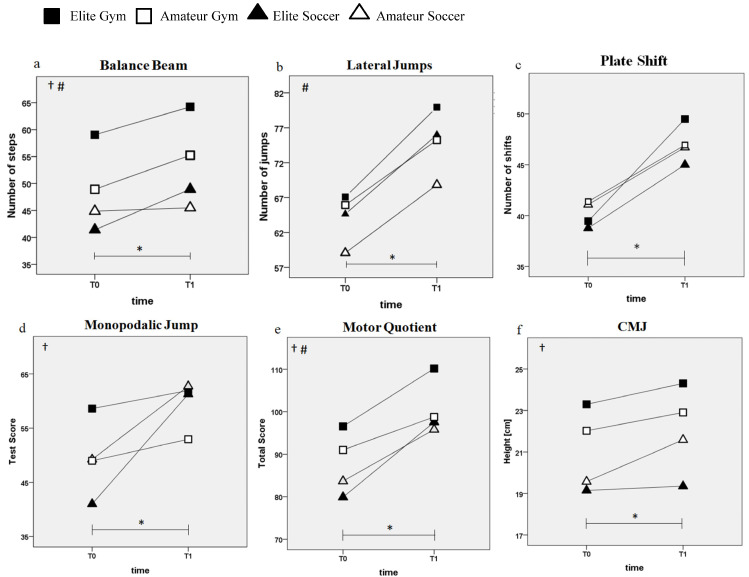
Physical test scores over time of the four groups: KTK: (**a**) Balance beam; (**b**) Lateral jumps; (**c**) Plate shift; (**d**) Monopodalic jump; (**e**) Motor quotient. (**f**) CMJ. Significant effects of * time; † time × sport interaction; ^#^ time × level interaction.

The linear mixed model reported significant effects of time and its interaction with sport and level, both in KTK battery and CMJ. There was a significant effect of time (F_(1,82)_ = 22.42, *p* < 0.001, ηp^2^ = 0.21), time × sport (F_(2,88.7)_ = 14.0, *p* < 0.001, ηp^2^ = 0.24), and time × level (F_(2,89.9)_ = 3.32, *p* = 0.041, ηp^2^ = 0.07) on BB values. Overall, athletes improved their balance over time, however, the amateur soccer group showed only a slight improvement (Figure 2a). In the LJ, a significant effect of time (F_(1,83) =_ 99.94, *p* < 0.001, ηp^2^ = 0.55) and time × level (F_(2,89)_ = 2.30, *p* = 0.024, ηp^2^ = 0.05) was observed, since all groups improved their agility over time, with a major increase in the elite groups (Figure 2b). In the PS test, the result showed a significant effect of time (F_(1,82.5)_ = 127.04, *p* < 0.001, ηp^2^ = 0.6) as all groups improved their test score at T1, with no significant interaction effects with sport or level (Figure 2c). In the MJ test, significant effects of time (F_(1,78.6)_ = 111.98, *p* < 0.001, ηp^2^ = 0.59) and time × sport (F_(2,85.8)_ = 24.34, *p* < 0.001, ηp^2^ = 0.36) were observed, as all groups improved over time in this test, with a greater rate of improvement in the soccer groups (Figure 2d). The total MQ score showed significant effects of time (F_(1,80.7)_ = 179.50, *p* < 0.001, ηp^2^ = 0.69), time × sport (F_(2,87.9)_ = 9.34, *p* < 0.001, ηp^2^ = 0.18) and time × level interactions (F_(2,88.1)_ = 5.68, *p* = 0.005, ηp^2^ = 0.11), as all groups improved over time, with a greater rate of improvement in the soccer and elite groups (Figure 2e). Finally, values of CMJ showed a significant effect of time (F_(1,77.9)_ = 15.86, *p* < 0.001, ηp^2^ = 0.17) and time × sport interaction (F_(2,85.7)_ = 3.86, *p* = 0.025, ηp^2^ = 0.08), as there was an overall improvement in CMJ height, with the greatest rate of improvement in soccer athletes (Figure 2f).

### 3.4. Executive Functions Differences over Time

**Figure 3:**
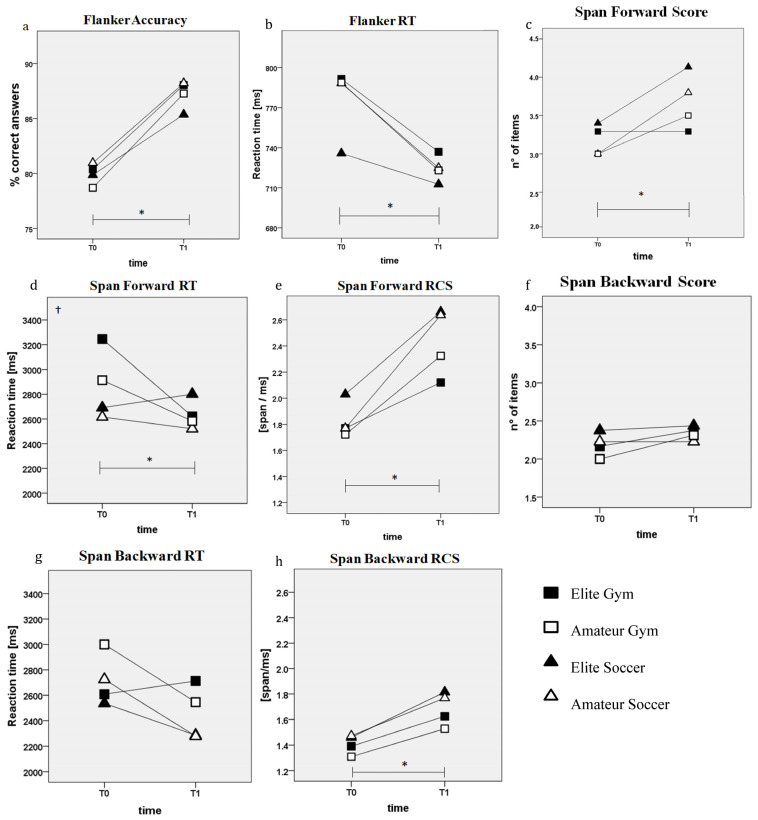
Executive functions scores over time of the four groups: (**a**) Flanker accuracy; (**b**) Flanker reaction time; (**c**) Digit Span forward score; (**d**) Digit Span forward reaction time; (**e**) Digit Span forward rate of correct score; (**f**) Digit Span backward score; (**g**) Digit Span backward reaction time; (**h**) Digit Span backward rate of correct score. Significant effects of * time; † time × sport interaction.

The linear mixed model reported significant effects of time and time × sport interaction, with no effects of time × level interaction on EFs tests. In particular, the results showed a significant positive effect of time on Flank_Acc (F_(1,78)_ = 75.92, *p* < 0.001, ηp^2^ = 0.49) and Flank_RT (F_(1,83.5)_ = 37.27, *p* < 0.001, ηp^2^ = 0.31) while no effects of sport, level, or their interaction were observed in this test (Figure 3a,b). Also, for the Span_for, significant effects were observed only for time (F_(1,81.5)_ = 11.60, *p* = 0.001, ηp^2^ = 0.12); however, while both soccer groups improved at T1, no improvements were observed in elite gymnasts, which remained stable over time (Figure 3c). Significant positive time (F_(1,75.7)_ = 6.78, *p* = 0.011, ηp^2^ = 0.08) and time × sport (F_(2,80.4)_ = 4.11, *p* = 0.020, ηp^2^ = 0.09) effects were found in the Span_for_RT, as a constant improvement was observed over time in gymnastics groups compared to soccer athletes (Figure 3d). No significant effects of the fixed factors analysed, or their interaction, were observed in the Span_back (Figure 3f), and Span_back_RT (Figure 3g) while the significant positive effect of time was observed in the RCS of Span Digit both forward (F_(1,77.8)_ = 35.61, *p* < 0.001, ηp^2^ = 0.31) and backward (F_(1,83.9)_ = 14.64, *p* < 0.001, ηp^2^ = 0.15) (Figure 3e,h).

### 3.5. Correlation Analysis Between Motor Performance and EFs

Overall, Spearman’s correlation coefficient showed several significant correlations between GMC and EFs. In particular, Flank_Acc, Span_back, and Span_RCS (both forward and backward) showed weak positive correlations with all (except for BB) GMC tasks and the MQ total score. Moreover, Flank_RT showed a moderate negative correlation with LJ and a weak negative correlation with PS and the MQ total score (a negative ρ coefficient indicates a positive relationship between RT ability and coordinative tests). Span_for showed a weak positive correlation with LJ while no significant correlations were observed for CMJ and EFs performance. Spearman’s correlation coefficients between cognitive and motor performance are reported in Table 4.

## 4. Discussion

### 4.1. Motor Performance in Gymnastics and Soccer Athletes

Gymnastics athletes showed better scores in GMC and explosive lower limb power than soccer athletes. Results of superior balance ability observed in gymnasts compared to athletes from other sports are not novel [16,17,18,19,20,21,22,23,24,25,26,27,28,29,30,31,32,33,34,35,36,37,38,39,40,41]. Gymnastics training involves a high degree of body control and proprioceptive awareness, which may also explain the superior balance abilities found in the present study. Even if the walking backward test of the KTK battery could seem to be gymnastics-specific, similar results were observed also in less movement-specific balance tests. Kenville et al. [42] observed a significantly greater level of balance in male gymnasts compared to soccer athletes (9–12 years) as indicated by the shorter centre of the pressure path length of the one-legged stance test. In disagreement with us, Bressel et al. [43] found no differences in balance abilities between gymnasts and female soccer athletes. The results could be explained by the subjects’ age: in fact, they compared college athletes (around 21 years old), a phase of growth where the development of coordinative abilities had already reached its peak [44].

In addition to balance, gymnasts showed greater values on all jump tests compared to soccer athletes. These results were not so predictable, considering that the MJ and CMJ involve lower limb power and coordination, which are components of both soccer and gymnastics sports activities. Contrary to our results, Formenti et al. [19] found that children (10–11 years) practicing OSS (including soccer) showed better agility compared to peers practicing CSS (including gymnastics) and greater CMJ height compared to sedentary children (while the CSS did not). The positive results of the jumping and agility test of the gymnasts of this study could be likely explained by their anthropometric characteristics. Based on this study’s samples, gymnasts have a significantly lower BMI compared to soccer athletes (gymnastics: 17.9 ± 2.2 kg/m^2^ vs. soccer: 19.9 ± 4.0 kg/m^2^), that could have increased their height in jumping and hopping tasks as a result of greater relative strength and power [45]. In the PS test no differences have been found between groups, possibly due to the hand-eye coordination required in the task. Indeed, although gymnastics involves significantly more upper-body engagement compared to soccer, there is no use of small apparatus, which could have provided an advantage in upper-body object-control tasks, as seen in rhythmic gymnasts [46].

Overall, the high positive impact on GMC in gymnasts is in line with existing literature: Wang et al., in a recent meta-analysis, reported that children involved in motor-development exercise (including gymnastics, rhythmic activities and music use) showed greater levels of GMC compared to peers involved in ordinary PA [47]. However, in other studies, children practicing OSS, like soccer, showed superior levels of MC compared to children practicing CSS [19]. Spanou et al. [16], suggested that MC development is influenced by the type of sport practice (team/individual—and OSS/CSS) as it differentiates the motor skills of children practicing a specific sport. Nevertheless, in agreement with us and previous research [10], the authors reported that children practicing individual CSS (like gymnastics) had the highest total motor proficiency score compared to the other categories [16].

Analysing sports groups of the same level, we observed that these differences in GMC were still present when comparing the elite groups (elite gym vs. elite soccer) but disappeared when comparing the amateur groups (amateur gym vs. amateur soccer). Moreover, no differences in GMC were found between elite and amateur soccer athletes. Thus, based on the results of this study, we can assert that is not the sport itself, but the combination of the sport and level, which could promote a greater GMC development in preadolescent female children. In fact, playing at the elite level has been previously associated with a higher level of motor coordination in preadolescents [25].

In the longitudinal section we found that there was a main positive effect of time on motor performance, as all groups improved in all motor tests. This result is in accordance with the correlation between age and motor development, as, even in a nonlinear manner, they are positively associated [2]. In addition, some sport and level effects were also observed in the analysis over time. Specifically, in accordance with the literature [41,42], the improvement in BB was more pronounced in elite gymnasts. Moreover, the jumps improvement was higher in soccer groups, while the final MQ improvement was higher in soccer and elite groups. These results are in line with the previous literature reporting that preadolescent soccer athletes significantly improved their lower limb power in a period of only four weeks of soccer training [48]. However, it is worth highlighting that in our study, despite soccer athletes showing higher improvement over time in some tests, the absolute values of all motor tests remain highest in elite gymnasts at the end of the sport season (Figure 2). These results represent a description of motor skill progression over time, as the lack of a control group prevents isolating the influence of other key factors, such as natural motor development.

### 4.2. Executive Functions in Gymnasts and Soccer Athletes

The results of EFs analysis showed that soccer athletes have a better RT in the WM task, compared to gymnasts. Moreover, considering the different levels of expertise, elite soccer athletes had better RT in both IC and WM tasks compared to both amateur soccer athletes and elite gymnasts. This difference in EFs level, like motor skills results, was not observed comparing elite vs. amateur gymnasts, not even among amateur groups themselves.

Even though the potential mechanisms underlying this positive effect of OSS on EF development remain unclear, the causes of these benefits could lie in the higher cognitive load required in OSS. In their review, Gu et al. [13] suggested that sports involving continuous adaptation to a changing environment (such as soccer) require visuospatial abilities, information-processing speed, and other core EFs. This could reflect the general increase in cognitive load in OSS compared to CSS, thus providing additional benefits for children’s cognitive development during a period of brain maturation [7,8]. In technical-combinatory sports like gymnastics, the low variability due to the continuous skills repetition and refinement for movement automatization may account for the lower total cognitive involvement.

In agreement with our results, several authors found a positive association between EFs levels and team OSS [19,49,50]. Specifically, higher levels of IC have been observed in children practicing OSS compared to both sedentary and CSS groups [19]. Waellae et al. found that children practicing team sports have greater EFs levels compared to both children practicing individual sports or sedentary female peers [49]. With a study sample comparable to ours, Bidzan-Bluma et al. [1] found that children (10–12 years) practicing soccer had better cognitive functions (attention and cognitive flexibility) compared to gymnasts. Contrary to our results, gymnasts showed better verbal WM, however, the same positive results were not achieved when a visual WM task (similar to the WM test used in our study) was employed. The authors also reported that both soccer and gymnast athletes had higher cognitive functioning compared to a control group [1]. In our study, we did not include a control group, as previous research has already demonstrated the positive effects of sport intervention in the cognitive domain [1,11,19,51].

Regarding athletes’ sport levels, the superior EFs levels of elite soccer athletes compared to amateur soccer groups found in this study seem to agree with the existing literature [50]. Indeed, higher levels of IC have been observed in elite soccer athletes [23] and table tennis players [52] compared to the sub-elite groups of their respective sports. Moreover, some authors suggested that a high EFs degree may discriminate between low-level and high-level athletes in both volleyball [53] and soccer [54]. Sakamoto et al. [54] analysed the EFs of 383 male soccer players (8–11 years old) applying for admission to an elite soccer program. The authors found that athletes selected for the elite soccer program had significantly better EFs scores compared to the rejected players [54]. Other authors [55] found that adolescent soccer players who demonstrated superior tactical performance had a shorter RT in IC tests compared to peers with lower tactical performance. The ability to inhibit inappropriate tactical decisions and to quickly respond to a stimulus by identifying and selecting relevant information has been considered important for players’ success in team sports [17]. Nevertheless, the question regarding the direction of this relationship remains open. Further studies are needed to understand whether experience in OSS leads to higher EFs levels, or if having a superior EFs level enhances sports performance. Analysing these results, it could be inferred that the increase in EFs level of elite soccer players could be attributable to the greater time spent doing sport. However, elite soccer athletes also outperformed elite gymnasts in RT of both IC and WM tasks, despite the comparable amount of weekly training time. Soccer training generally involves a high variability of movements, technical skills, strategic thinking [56] and sport-specific situational exercises, whereas technical-combinatory sports like gymnastics are based on a gradual process of new skill acquisition requiring an extended period of time. Thus, with a similar amount of weekly training hours, cognitive involvement appears to be more prominent during soccer practice sessions, while the effects of sport practice on brain activity in gymnasts are likely to become evident after extended periods of intensive training [57].

Contrary to these findings, Spanou et al. [16] found that children involved in OSS (both team and individual) or CSS showed comparable levels of IC and cognitive flexibility [16]. In addition, Holfelder et al. [24] also did not find a significant effect of the type of sport on the Flanker Task in handball and track and field young athletes (mean age 14 years old). An explanation of these results might depend on another factor that could be associated with EFs development: aerobic exercise. In fact, previous research has highlighted the role of aerobic training in EFs development [11,12,13,14,15,16,17,18,19,20,21,22,23,24,25,26,27,28,29,30,31,32,33,34,35,36,37,38,39,40,41,42,43,44,45,46,47,48,49,50,51,52,53,54,55,56,57,58] that is strongly present in both open-skill and track and field sports, included in both the cited studies. In this study, we can exclude this third factor, as artistic gymnastics is a sport that predominantly relies on anaerobic metabolic pathways.

The results of EFs changes over time show that time had a main significant effect on all analysed parameters of IC and WM. These results are in line with previous studies highlighting the great impact of age on EFs development [16,17,18,19,20,21,22,23,24,25,26,27,28,29,30,31,32,33,34,35,36,37,38,39,40,41,42,43,44,45,46,47,48,49,50,51,52,53,54,55,56,57,58,59], as improvement over time has been observed in both open- and CSS [12] and even only after 4 weeks of training [48]. Additionally, both elite and amateur soccer players showed an improvement in WM by the end of the sports season, whereas gymnasts showed a higher improvement in the Forward-Digit Span RT, approximately reaching the values of soccer groups, recovering the gap with their soccer peers. Overall, the changes observed over the sport season did not seem to be associated with a specific sport or a level (no effect of level per time interaction was observed). Moreover, the absence of a same-age sedentary group, prevents us from distinguishing the potential benefits of OSS or CSS on the rate of EFs development. In accordance with our findings, previous authors of longitudinal studies have concluded that the developmental trajectories of EFs in both gymnasts [60] and soccer athletes [61] are similar to those in the general population, which also improve rapidly during childhood.

### 4.3. Correlations Between Motor and Cognitive Functions

In this study, only weak positive correlations have been found between GMC and EFs. A moderate association was only observed between the Flanker RT and the agility motor task (LJ). These results are in line with previous research which found only a weak association between EFs tests and GMC in female children (9–10 years old) using the same motor assessment battery [62]. Moreover, a recent review on the association between EFs and motor competencies in children, concluded that only small-to-moderate positive associations exist between motor competence and EFs in this population [63]. In this study, higher values of both EFs and motor skills were primarily observed in elite athletes, which could lead to the misleading hypothesis that these are significantly correlated due to a higher degree of expertise. However, the fact that elite gymnasts demonstrated high levels of physical skills but lower EFs levels, whereas elite soccer players show high EFs scores but lower physical skills, may explain why the correlations between motor and cognitive functions are weak. Therefore, despite our sample consisting of children who regularly engage in sports, it did not show a stronger correlation compared to the general population of comparable age.

### 4.4. Limitations of the Study

A study limitation concerns the sample selected that was limited to female children. However, previous research has pointed out that no significant gender differences were found in either motor competencies or EFs among children practicing open- or CSS [16]. Nevertheless, a deeper analysis including also male subjects could provide additional information on this topic. In relation to the relatively small sample size (considering multiple comparisons between groups), only medium-to-large differences between groups were detected. Thus, smaller variations, which would require a larger sample size for detection, were not identified, and this should be considered when interpreting these findings. A further limitation is the duration of the longitudinal study and the lack of a control group. To gain a more comprehensive understanding of the growth trajectories of both motor and cognitive functions a longer follow-up period and the presence of a control group could have provided additional insights.

## 5. Conclusions

In conclusion, this study demonstrated that female children participating in a closed-skill and technical-combinatory sport (artistic gymnastics) exhibit higher levels of GMC compared to athletes practicing an open-skill team sport (soccer). On the other hand, soccer athletes showed higher executive function levels compared to their gymnastics counterparts. However, caution should be used when interpreting these findings, since a great influence of the athletes’ level of expertise was observed. Indeed, it is not only the sport itself but also the association between the sport and the level of expertise, which showed the most significant results. In fact, the primary contribution to the observed differences in sports performance was due to the scores of the elite groups, while the amateur groups alone did not show significant differences in either motor or cognitive domains. Thus, in agreement with previous research [64], it can be deduced that the impact of PA on executive functions does not necessarily involve alterations in GMC, nor vice versa. Considering that the practice of sports requiring high cognitive demands during childhood can contribute to the development of executive functions throughout growth [65], the results of this study may prove valuable in selecting sports disciplines for children with specific developmental needs, both in motor and cognitive domains.

## Figures and Tables

**Table 1:** Weekly training programs of gymnastics and soccer groups.

	Elite Gym	Amateur Gym	Elite Soccer	Amateur Soccer
Average weekly workout session frequency [n/week]	4 ± 1	2 ± 0	3 ± 0	2 ± 0
Average duration of a single workout session [min]	115 ± 30	90 ± 0	120 ± 0	75 ± 0
Average weekly training hours * [h]	7.5 ± 0.5	3.3 ± 0	7.5 ± 0	3.7 ± 0.5

* For soccer groups: training + matches.

**Table 2:** Anthropometric data of gymnastics and soccer groups.

	Elite Gym	Amateur Gym	Elite Soccer	Amateur Soccer
Weight [kg]	37.7 ± 6.3	38.2 ± 10.0	46.9 ± 10.3 *	42.7 ± 11.6
Height [cm]	144.8 ± 6.4	144.5 ± 9.1	149.3 ± 8.1	148.0 ± 7.3
BMI [kg/m^2^]	17.9 ± 1.9	18.0 ± 2.6	20.9 ± 3.9 *	19.3 ± 4.0

* *p* ≤ 0.05; Significant different from elite gym.

**Table 3:** Baseline values of physical and cognitive tests of gymnastics and soccer groups.

	Elite Gym	Amateur Gym	Elite Soccer	Amateur Soccer
BB [n° of steps]	58.31 ± 10.54 **^##^**	48.53 ± 10.01 **	41.71 ± 12.75	44.73 ± 10.26
LJ [n° of jumps]	66.60 ± 7.88	65.06 ± 9.25 **^#^**	64.76 ± 12.28	58.42 ± 11.18
PS [n° of shifts]	39.92 ± 6.14	40.71 ± 5.64	38.76 ± 5.10	41.39 ± 4.80
MJ [score]	58.64 ± 10.36 **^##^**	48.59 ± 11.38 **	42.29 ± 8.19	51.09 ± 11.98 **
MQ [score]	96.16 ± 8.44 **^##^**	90.24 ± 10.91	80.57 ± 10.18	84.21 ± 12.03
CMJ [cm]	22.86 ± 4.31 **^#^**	21.76 ± 4.66	19.48 ± 4.48	20.02 ± 3.73
Flank_Acc [%]	80.73 ± 9.65	78.41 ± 10.24	81.11 ± 9.73	80.10 ± 11.31
Flank_RT [ms]	789.14 ± 70.68 **^#^**	791.28 ± 91.27	734.74 ± 105.91	788.61 ± 71.27 *
Span_for [n° of items]	3.31 ± 1.23	3.00 ± 1.60	3.38 ± 1.16	3.04 ± 1.15
Span_for_RT [ms]	3181.93 ± 1372.82	3256.27 ± 1503.91	2714.83 ± 534.77	2688.26 ± 553.80
Span_for_RCS [span/ms]	1.80 ± 0.70	1.65 ± 0.83	1.99 ± 0.85	1.80 ± 0.62
Span_back [n° of items]	2.15 ± 0.78	2.00 ± 1.32	2.19 ± 0.98	2.36 ± 1.06
Span_back_RT [ms]	2566.34 ± 1051.07 **^#^**	2937.77 ± 1664.65	2318.16 ± 1223.38	2798.33 ± 1121.63 *
Span_back_RCS [span/ms]	1.40 ± 0.51	1.35 ± 0.61	1.52 ± 0.57	1.49 ± 0.53

* *p* ≤ 0.05; ** *p* ≤ 0.01 Amateur gym vs. elite gym and amateur soccer vs. elite soccer. **^#^** *p* ≤ 0.05; **^##^** *p* ≤ 0.01 Elite gym vs. elite soccer and amateur gym vs. amateur soccer. Table legend. BB: balance beam; LJ: lateral jumps; PS: plate shift; MJ: monopodalic jumps; MQ: motor quotient; CMJ: countermovement jump; Flank_Acc: Flanker accuracy; Flank_RT: Flanker reaction time; Span_for: Digit Span forward; Span_for_RT: Digit Span forward reaction time; Span_for_RCS: Digit Span forward rate of correct score; Span_back: Digit Span backward; Span_back_RT: Digit Span backward reaction time; Span_back_RCS: Digit Span backward rate of correct score.

**Table 4:** Correlations between EFs and motor performance.

Spearman (ρ)	Flank_Acc	Flank_RT	Span_For	Span_for_RT	Span_for_RCS	Span_Back	Span_Back_RT	Span_Back_RCS
BB	0.234 **	−0.004	−0.034	−0.110	0.096	0.149 *	0.094	0.047
LJ	0.388 **	−0.438 **	0.207 **	−0.106	0.294 **	0.244 **	−0.010	0.306 **
PS	0.343 **	−0.275 **	0.118	−0.084	0.156 *	0.155 *	0.034	0.163 *
MJ	0.379 **	−0.133	0.124	−0.006	0.173 *	0.216 **	0.073	0.155 *
MQ	0.390 **	−0.227 **	0.108	−0.020	0.156 *	0.228 **	0.101	0.175 *
CMJ	0.118	−0.075	0.074	0.041	0.035	0.057	0.010	0.099

* Significant ρ correlation *p* < 0.05; ** significant ρ correlation *p* < 0.01.

## Data Availability

Data are available upon request to the contact author.
